# Zika Virus Infection and Microcephaly

**DOI:** 10.15844/pedneurbriefs-30-1-7

**Published:** 2016-01

**Authors:** J. Gordon Millichap

**Affiliations:** 1 Division of Neurology, Ann & Robert H. Lurie Children's Hospital of Chicago, Chicago, IL; 2 Departments of Pediatrics and Neurology, Northwestern University Feinberg School of Medicine, Chicago, IL

**Keywords:** Zika virus, Microcephaly, Brazil, Aedes mosquitoes

## Abstract

A Task Force established by the Brazil Ministry of Health investigated the possible association of microcephaly with Zika virus infection during pregnancy and a registry for microcephaly cases among women suspected to have had Zika virus infection during pregnancy.

A Task Force established by the Brazil Ministry of Health investigated the possible association of microcephaly with Zika virus infection during pregnancy and a registry for microcephaly cases among women suspected to have had Zika virus infection during pregnancy. In early 2015, an outbreak of Zika virus, a flavivirus transmitted by Aedes mosquitoes, was identified in northeastern Brazil. By September 2015 an increase in the number of infants born with microcephaly began to emerge, and Zika virus RNA was identified in the amniotic fluid of 2 women with fetuses having microcephaly on ultrasound. Among a cohort of 35 infants with microcephaly born during August-October 2015 in eight of Brazil’s 26 states and reported to the registry, the mothers of all 35 had lived in or visited Zika virus-affected areas during pregnancy, 25 (71%) infants had severe microcephaly (head circumference >3 SD below the mean for sex and gestational age), 17 (49%) had at least one neurologic abnormality (hypertonia/spasticity [37%], seizures [9%]), and among 27 infants who had neuroimaging studies, all had abnormalities (brain calcifications [74%], ventricular enlargement [44%], neuronal migration disorders [33%]). Tests for other congenital infections were negative. CSF tests for Zika virus infection are not yet available, and further studies are needed to confirm the association of microcephaly with Zika virus infection during pregnancy. [1]

COMMENTARY. Microcephaly is defined as a head circumference -/= SD below the mean for sex and gestational age at birth [2]. Except in cases of craniosynostosis, a small skull reflects a small brain. Microcephaly is either primary (anomaly of development during the first 7 months of gestation) or secondary to an insult incurred during the last 2 months of gestation or during the perinatal period. CDC has developed interim guidelines for health care providers in the US caring for pregnant women during a Zika virus outbreak. Pregnant women with a history of travel to an area with Zika virus transmission (as of Jan 2016, 19 countries in the Americas outside Brazil) and who report two or more symptoms of Zika virus disease (acute onset of fever, maculopapular rash, arthralgia, or conjunctivitis) during or within 2 weeks of travel, or who have ultrasound findings of fetal microcephaly or intracranial calcifications, should be tested for Zika virus infection. In pregnant women with laboratory evidence of Zika virus infection, serial ultrasound should be considered to monitor fetal growth [3]. No specific antiviral treatment for Zika virus is available.
